# Exploring influences on evaluation practice: a case study of a national physical activity programme

**DOI:** 10.1186/s12966-021-01098-8

**Published:** 2021-02-16

**Authors:** Judith F. Fynn, Wendy Hardeman, Karen Milton, Andy Jones

**Affiliations:** 1 UKCRC Centre for Diet and Activity Research (CEDAR) and Norwich Medical School, University of East Anglia, Norwich, UK; 2 School of Health Sciences, University of East Anglia, Norwich, UK; 3 Norwich Medical School, University of East Anglia, Norwich, UK

**Keywords:** Physical activity, Evaluation, Evidence-based public health, Influences on practice

## Abstract

**Background:**

Interventions to improve physical activity behaviour are a core part of public health policy and practice. It is essential that we evaluate these interventions and use the evidence to inform decisions to improve population health. Evaluation of ‘real-world’ interventions provide an opportunity to generate practice-relevant evidence, however these interventions are difficult to evaluate. Various guidelines have been developed to facilitate evaluation, but evidence about their effectiveness in practice is limited. To explore influences on evaluation practice in an applied context, we conducted a case study of Sport England’s ‘Get Healthy Get Active’ (GHGA) programme. This was a national programme that funded 33 projects that were delivered and evaluated across England. The programme was chosen as it was designed to generate evidence on the role of sport in increasing physical activity and improving health. The study aimed to explore and appraise whether strategies intended to facilitate project evaluation, including funder requirements to use a standardised evaluation framework and specific data collection methods, were effective in generating evidence that enabled the programme to meet its aims.

**Methods:**

We applied a collective case study design involving 35 semi-structured interviews, and documentary analysis of multiple sources of evidence from 23 physical activity projects funded by GHGA. We applied thematic and framework analysis. We developed a logic model and mapped actual outcomes against intended outcomes. A narrative synthesis is provided. We discuss implications for the effective commissioning and evaluation of public health interventions.

**Results:**

We identified five main themes of influences on evaluation practices that can act as barriers and facilitators to good practice: programme and project design; evaluation design; partnerships; resources; and organisational structures and systems. These influences are context-specific and operate through a complex set of interactions.

**Conclusion:**

Developing a better understanding of how influences on evaluation practice can act as facilitators or barriers is vital to help close current gaps in the evidence-based practice cycle. Critically, organisational structures and systems are needed to facilitate collaborative decision making; integration of projects and evaluation across partners organisations; transfer of knowldege and insights between stakeholders; and more rapid feedback and dissemination.

**Supplementary Information:**

The online version contains supplementary material available at 10.1186/s12966-021-01098-8.

## Background

Interventions to increase physical activity are a core part of public health policy and practice [1–4], yet the complexity of public health interventions, which are often multi-component and multi-sectoral, inevitably leads to complexity in terms of their implementation and evaluation [5, 6]. Nevertheless, it is essential that we understand if and how these interventions are effective and act upon this evidence if we are to meet targets for increasing physical activity at the population level, including the World Health Organization Global Action Plan target for a 15% reduction in physical inactivity by 2030 [1].

Evidence-based public health aims to ensure that decisions and interventions are based on sound evidence to safeguard and improve the health of the population. Appropriate evaluation is central to the generation of this evidence [7–10]. One of the key challenges is to generate practice-relevant evidence, where external validity and adoption into routine practice may be more likely [10–12]. Evaluation of ‘real-world’ interventions, implemented as part of normal service delivery or in practice-based settings rather than in a research environment, provides an opportunity to address this challenge. However, this type of evaluation requires careful selection of approaches that are appropriate and feasible within real-world contexts [13–15].

Much progress has been made within the field of public health evaluation in the last two decades, and we have a better understanding of the challenges. Examples include limitations in expertise, capacity, and resources within normal service delivery to conduct evaluation, too much focus on operational objectives and outputs, and barriers to knowledge translation [7, 16–19]. As our understanding of the challenges to evaluation has developed, so too has the guidance available. This includes guidance on methodological approaches, such as theory-based or realist evaluation [20, 21], recommendations for good practice [8, 14, 16, 22–24], and specific frameworks to facilitate systematic evaluation [25–27]. The application of frameworks and logic models are now commonly recommended to guide the evaluation and reporting of physical activity interventions. However, our own systematic review of evaluation frameworks showed limited use and/or reporting of frameworks in evaluation studies of physical activity interventions [28]. The reasons for this remain unclear.

Further to the concerns regarding the limited use of frameworks, additional gaps remain in our understanding of how to improve evaluation. Previous reviews of health promotion programmes have highlighted a need for a greater consideration of programme theory [29], investment and planning for evaluation [7], and a need for multi-level strategies that involve multiple stakeholders [7, 16, 19]. Collaboration with independent experts in evaluation, such as through research-practice partnerships, is recommended as an approach to improve the quality of evaluation, build capacity for evaluation [7, 16, 18, 19, 22], and improve the use of evidence to inform programme development [12]. However, our understanding of the effectiveness of these strategies in practice remains limited [12, 19, 30, 31].

There is a need for research to develop a better understanding of how different factors interact to influence evaluation practice [19]. Lack of insight into these influences may lead to variability in the quality of evaluation and reporting, which limits the generation and use of critical evidence to inform interventions and decisions to improve population health.

In this study, we report the findings of a case study of Sport England’s ‘Get Healthy Get Active’ (GHGA) programme [32] to explore evaluation practices, and influences on practice, in an applied context. Sport England is the agency in England with primary responsibility for developing grassroots sports and increasing physical activity across England [33]. The GHGA programme was chosen as our case study as it was specifically designed to build an evidence base for the role of sport in increasing physical activity, improving health and reducing health inequalities [34]; evaluation was therefore a key element of the programme. The GHGA programme exemplifies multi-sectoral and multi-component approaches within public health [2]. We explored the relationships between organisational structures and processes, and evaluation practice. Although we focus on a national programme to increase physical activity, the aim was to produce research findings that were applicable to other health-promotion interventions, particularly those operating in multi-sectoral public health contexts.

## Objectives

1. To identify the logic of the programme and explore the relationships between programme and project aims.
2. To explore influences on evaluation practices, including requirements to use a standardised evaluation framework and specific data collection methods.
3. To appraise whether the programme was effective in generating high quality generalisable evidence that enabled it to meet its aims.
4. To formulate and discuss implications for the effective commissioning and evaluation of public health interventions.

## Method

### The GHGA Programme

Through the GHGA programme Sport England funded 33 physical activity projects, 31 projects within two funding rounds and two invited projects, which were delivered between 2013 and 2018 to communities and population groups across England. For clarity, we refer to the GHGA intervention as “the programme” and local, funded interventions as “projects”. Projects were developed, implemented and evaluated in partnership with Local Authorities, charities, Clinical Commissioning Groups and evaluation partners.

The programme provided an opportunity to explore evaluation practices, and to appraise whether strategies intended to facilitate project evaluation were effective. Sport England put in place several funding requirements to support evaluation. All projects were required to engage an independent evaluation partner, either an academic organisation or consultant. Projects were also required to use validated evaluation tools. This included the use of the Standard Evaluation Framework for physical activity interventions (SEF) [26] to guide project evaluation, the Single Item Physical Activity Measure [35], a validated tool to screen participants for eligibility for physical activity interventions, and the International Physical Activity Questionnaire (IPAQ) [36] to measure physical activity at baseline and follow-up.

### Study design

We applied a collective case study design [37], using documentary analysis and semi-structured interviews, to conduct an in-depth analysis of multiple sources of evidence from a range of physical activity projects funded by GHGA. Ethical approval was received from the University of East Anglia Faculty of Medicine and Health Sciences Reseach Ethics Committee (REF: 201718–133).

### Sampling and data collection for the documentary analysis

Agreement to conduct the research was gained from Sport England. We conducted initial screening of documents provided by Sport England or published on their website, such as the “Project Summaries”, to develop an overview of projects and to identify the lead organisation for each project. Each of the organisations responsible for the 31 projects in the two funding rounds were contacted and asked to share the final project evaluation report along with documents related to the funding application and intervention planning if available. Contact was initially made by email and then by telephone up to three times. All documents were given a unique code to de-identify them prior to importing them into NVivo 12 Pro for analysis.

### Sampling and data collection for the semi-structured interviews

For the interviews, we applied purposive sampling to select stakeholders who were involved in the development, delivery or evaluation of the GHGA programme and projects. This included stakeholders with a role in the national programme and the project lead of each organisation who had shared an evaluation report. We applied snowball sampling to identify additional stakeholders, such as evaluation partners and project facilitators. Each stakeholder was contacted up to three times via email or telephone and invited to participate in an interview. We continued sampling until we were confident that the sample was representative of projects across the two funding rounds, and different types of lead organisation, evaluation partnership, and stakeholder role. All participants provided written consent prior to participating in the interview.

We used semi-structured interviews to ensure we obtained data in relation to the objectives yet allow flexibility that may elicit richer data. An interview guide was developed to facilitate practitioner reflection and allow clarification of findings from the documentary analysis. The guide was piloted with one practitioner, however using semi-structured interviews allowed us to be responsive to emerging findings and refine the questions throughout the data collection period in an iterative approach. The guide consisted of 13 open ended questions that explored practitioners’ experiences of the evaluation process, influences on evaluation, barriers and facilitators, and dissemination activities (provided in Additional file 1).

The interview guide was sent to participants in advance to provide them with prompts for reflection prior to the interview. Interviews were conducted face-to-face, by Skype or telephone. One participant communicated their responses via email. Interviews were conducted by the lead author (JF) between May and December 2019 and lasted an average of 46 min (range 25–86 min). Interviews were audio recorded and transcribed verbatim. All transcripts were sent to participants to check and provide the opportunity to add additional comments or clarification. Transcripts were given a unique numerical identifier to de-identify them before being imported into NVivo12 Pro.

### Analysis of documents and interview data

To understand the programme aims and logic (objective one) we analysed Sport England’s organisational documentation related to programme design, funding and monitoring, to develop a logic model and pathway diagram. These were refined through interviews and consultation with key stakeholders at Sport England to ensure that our interpretation and representation of the programme was accurate.

To address objectives two and three we applied Framework Analysis [38, 39]. We combined deductive (a priori*)* and inductive (emergent) approaches to conduct thematic analysis of the documents and interview data. Initial categories and codes were identified a priori*.* These included codes related to the use and reporting of the SEF criteria, the single-item physical activity measure and the IPAQ. The SEF provides a structured framework to support project design, evaluation and reporting; the 52 criteria included in the SEF are intended to provide guidance on the information required to undertake a comprehensive and robust evaluation [26]. The criteria are grouped into seven sections (Table 1). We used these criteria as codes to guide data extraction and anaylsis, and provide a systematic approach to summarise the projects and their evaluation. Other codes identified a priori were informed by our interview guide and research objectives, for example influences on evaluation design, barriers and facilitators, and dissemination. Through repeated reading and familiarization with the data emergent codes were added, for example reference to additional evaluation methods such as logic models and case studies. The codes were reviewed and organised into categories and sub-themes (by JF) to develop the coding framework and were iterated and agreed with all authors.

**Table 1:** Summary of criteria included in the Standard Evaluation Framework for Physical Activity Interventions (SEF)

SEF sections	Criteria	Examples of criteria included
1 Programme details	16 essential 7 desirable	Aims, timescales, location and setting, description, recruitment, costs, resources Rationale, policy context, health needs assessment
2 Evaluation details	2 essential	Evaluation design, methods and timing of data collection
3 Demographics of participants	5 essential 2 desirable	Age, sex, ethnicity, disability, socio-economic status Additional information
4 Baseline data	1 essential 2 desirable	Measures of physical activity Correlates of physical activity, other outcomes
5 Follow up data	1 essential 3 desirable	Physical activity at ≥3 time points Physical activity > 1 year, correlates of physical activity, other outcomes
6 Process evaluation	6 essential 2 desirable	Participant numbers invited, recruited, attending, at follow up, satisfaction Unexpected outcomes, sustainability plans
7 Analysis & interpretation	3 essential 2 desirable	Summary of results, limitations and generalisability, recommendations Details of analysis, dissemination

We extracted data from NVivo12 Pro into a final analytical framework matrix to systematically synthesise the data by cases and codes. Using the framework we analysed themes by individual cases (funded projects), across different data sources (documents and interviews), and across the whole data set (representing the programme). To explore how evaluation practices had been applied and documented, and to identify influencing factors, we combined data from the documentary anaysis with data from the interviews.

The findings are presented as a narrative synthesis. Firstly, we present the programme’s aim and logic, and then describe how these compare to project aims and characteristics (objective 1). We then present key themes identified as influences on evaluation practices (objective 2). To appraise whether the programme aim of generating evidence had been met (objective 3), we summarise the reported outputs and outcomes from the project and programme evaluation, and map these against the intended outcomes. Finally, we formulate and discuss implications for effective commissioning and evaluation of health promotion interventions (objective 4) within the discussion.

## Results

### The case study sample

In addition to the programme-level documents provided by Sport England, representatives from 23 out of 31 (74%) projects shared documents, including the final evaluation reports. These documents formed our sample for the documentary analysis. Lead organisations of two projects declined to share reports, and the leads of the remaining projects did not respond, of which two organisations were known to be no longer in operation.

Thirty-five stakeholders participated in an interview, including stakeholders with a role in the development, management or evaluation of the national programme (*n* = 5), and stakeholders with a role in the design, delivery and/or evaluation of one or more local projects (*n* = 31). Some stakeholders had held more than one position with differing roles in the programme and projects. The interview sample was representative of 16 different projects; six from the first funding round and 10 from the second round.

### Objective one: to identify the logic of the programme and explore the relationships between programme and project aims

**Fig. 1:**
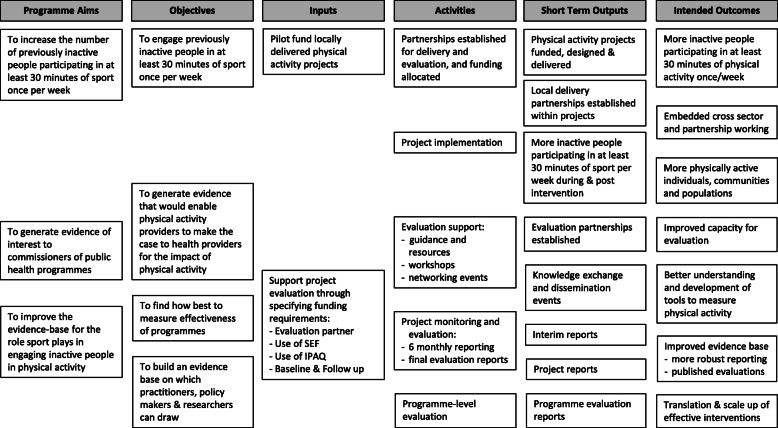
Logic Model for the Get Healthy Get Active (GHGA) programme

**Fig. 2:**
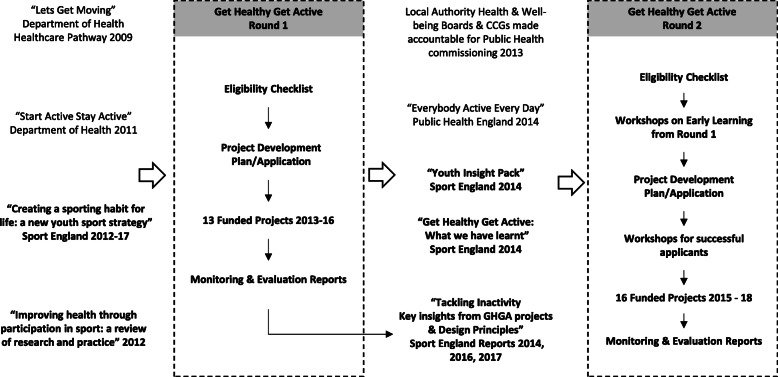
Pathway diagram of the Get Healthy Get Active (GHGA) programme. Notes: Round One was originally referred to as Get Healthy Get into Sport. Normal text shows external documents and influences on the programme e.g. Start Active Stay Active [40], Everybody Active Every Day [2], Bold text shows documents published or commissioned by Sport England and steps in the GHGA programme e.g. Sport England Strategy 2012–17 [41], Improving health through participation in sport [42], Get Healthy Get Active What we have learnt [34], Tackling Inactivity [43, 44]

The rationale for the programme and its evaluation is shown in a logic model (Fig. 1). A pathway diagram (Fig. 2) shows the contextual factors influencing the programme. The programme was described as a response to a review commissioned by Sport England that highlighted the limited evidence base for the role of sport in tackling inactivity [42], and to government strategies that sought to increase participation in sport and physical activity among the least active adults [40, 41]. Stakeholders involved in the programme’s design highlighted the desire to build evidence that could support the commissioning of sport interventions to improve physical activity and health. One programme-level stakeholder explained:

“The reason why we did it the way we did it, was because of the lack of the evidence base … so when somebody else does a systematic review we are hoping that there will be at least 33 papers that will come up, if not more, to help answer that question in future”. (stakeholder 1)

Table 2 summarises the aims and key characteristics of the projects. Whilst the primary aim of all projects aligned to the programme aims, projects also reported various secondary aims and objectives. Projects were delivered by a range of organisations and cross-sector partnerships in a range of locations and settings to diverse population groups. Several included multiple components and/or delivery pathways.

**Table 2:** Summary of the reported programme and project characteristics, aims and objectives

Project	Lead Organisation	Evaluation Partner	Location and Setting	Target Population	Aims and Objectives
GHGA	Sport England	In-house and independent consultants	NA	Inactive people aged 14 years and over	To encourage inactive adults to increase their physical activity by participating in sport, and build the evidence base
1–01	County Sports Partnership	University Partner	County-wide community settings	Inactive adults aged 16 years and over	How inactive adults can be recruited into sport and PA; How sport can be used to engage inactive adults in PA; Assess the impact and cost-effectiveness
1–02	University	University Led	CCG area, sport and leisure settings	Inactive people with hypertension, suspected or pre-hypertension or high-normal blood pressure	Whether sports-based referral for exercise would be effective compared to traditional gym-based projects; Whether a self-help web-based tool would add any additional benefit
1–03	University	University Led	Metropolitan borough, community settings	Inactive people	To design and deliver innovative community sports for health projects in different local contexts; Evaluate the design, outcomes, processes and costs of the project.
1–04	County Sports Partnership	University Partner	County-wide	Sedentary people at excess risk of cardiovascular disease and Type 2 diabetes	To describe the demographic details and impact of the project on self-reported and objectively measured physical activity; To gain insights into the experiences of participants and deliverers
1–05	County Sports Partnership Network	University Partner	National workplaces	Inactive employees	To develop a package of interventions to engage people in PA in workplaces; Assess the effectiveness of the project on increasing sport & PA and on business outcomes; Understand factors associated with using the workplace to engage the inactive in sport and PA
1–06	County Sports Partnership	University Partner	City and County districts, community settings	Inactive people living in target areas	To develop and test a community model for engaging inactive individuals in sport and PA; Assess whether one-to-one mentoring influences experiences and adherence to participation in sport and physical activity; Explore influences of engagement of family and friends; Explore wider benefits; Explore impact of engaging volunteers
1–07	Charity	Evaluation Consultant	Geographical Health regions across UK	People Living with Cancer	Understand how the pathway has been implemented; Assess the extent to which delivery is in line with the ideal model; Explore efficacy of the interventions, scalability of the pathway, processes for best practice delivery, and impact of the pathway on service users and their families
1–08	County Sports Partnership	University Partner	County-wide, leisure settings	Referrers of inactive people (various health services)	To help individuals meet recommended levels of physical activity, based on the Lets Get Moving pathway
1–09	County Council	University Partner	County-wide, community settings	Inactive adults with long-term health conditions: cancer, cardiovascular disease, type II diabetes, mental health and from deprived communities	To establish the effectiveness of the project at increasing and sustaining PA of inactive individuals; Establish the effectiveness of tailoring interventions to specific population groups; Understand the mechanisms by which outcomes were reached and identify good practice and difficulties
1–10	Not-for-profit association	Not Stated	City and County-wide, GP surgeries	Individuals 18–75 years with a BMI between 28 and 35 resident in the catchment of participating surgeries	To provide an overarching assessment of the project and its impact upon participation in sporting sessions and physical activity levels
1–11	Borough Council group	University Partner	Metropolitan borough	Inactive people aged 14 and over, with a BMI of 28 or more	To help people get fit and lose weight by taking up sport; Evaluate effects of a community sports referral project compared with standard community exercise referral
2–01	County Sports Partnership	University Partner	County-wide, sheltered housing and care homes	Residents aged 65 years and over in sheltered housing and care home sites	To promote physical activity among residents in group homes with the aim of normalising physical activity
2–02	Not-for-profit association	University Partner	County districts	Inactive people over 16 years, living in target areas, one or more risk factors for cardiovascular disease &/or mild to moderate mental health problems	To support inactive adults to become more active and to work with Primary Health Care as a primary route of referral; Assess the measurable change on PA, general health and wellbeing; Understand how the project worked
2–03	City Council	Evaluation Consultant	City areas, community settings	Pregnant and post-pregnant women	To increase the activity levels of pregnant and post-pregnant women
2–04	County Sports Partnership	University Partner	County-wide, leisure and community settings	People with drug and alcohol related problems	To encourage active and healthier lifestyles for adults recovering from drug and alcohol misuse
2–05	Borough Council	University Partner	Metropolitan borough, community settings	Inactive people with a high risk of developing type 2 diabetes, aged 47–74 years	To show the impact of a targeted sport & PA project on helping prevent or reduce the onset of type 2 diabetes and risk factors, for high risk adults; Assess differences across demographic categories; Assess if peer support can impact on someone increasing (and maintaining) PA; Assess differences in GP- and self-referred
2–06	Borough Council	University Partner	County-wide	Inactive people with a long-term condition: Cardiac Phase IV, Chronic Heart Failure, Stroke, Cancer, Lower Back Pain, Chronic Obstructive Pulmonary Disease & Falls Prevention	To support individuals with long term conditions to become and stay more physically active; To understand how effective the project was in providing condition specific support via PA pathways for seven long-term conditions, cost effectiveness, and the process of delivering the programme
2–07	Borough Council	University Partner	Metropolitan borough	Older adults	To engage inactive older adults in PA at least once a week for 30 min; Evaluate project effectiveness on older adults’ physical activity, sedentary behaviour and self-reported health indicators
2–08	District Council	University Partner	District, leisure & community settings	Inactive, hypertensive, pre-diabetic, diabetic or overweight/obese people	To engage individuals in sport and PA through collaborative working between general practice and community leisure services; Understand the population impact; Understand Reach, Effectiveness, Adoption, Implementation and Maintenance
2–09	Not-for-profit association	University Partner	Metropolitan borough, community settings	Residents	To support and empower residents to lead healthier lives, to be more active and lose/maintain a healthy weight
2–10	University	University Led	City-wide	Young people (14-25 yrs), working adults and older adults (65+), and those with an identified health risk through smoking or obesity	To put in place a city-wide (whole systems) approach to tackling physical inactivity; Investigate changes in PA awareness and behaviour in response to the implementation of a consortium-led, multi-agency, person-centred behaviour change project
2–11	County Council Public Health	Evaluation Consultant	County-wide, leisure and community settings	Inactive people in the County	To enable inactive people to engage with sporting activities to lower rates of physical and mental ill-health and to reduce public expenditure related to preventable illness; Evaluate how implementation has improved outcomes and experiences for participants, including improvements in quality of life, health and well-being
2–12	Not-for-profit association	University Partner	City-wide	Inactive men & women (aged 26–75) who already had type 2 diabetes or were pre-diabetic or were at high risk of type 2 diabetes	To engage target population in a community-based sport and PA intervention to increase PA, enhance health and wellbeing and facilitate the management of disease symptoms

The pathway diagram (Fig. 2) shows changes in organisational structures and strategies, as well as organisational learning [34, 34, 43], which influenced programme processes and practices across the two funding rounds. A key factor was the shift to Local Authority Health and Well-being Boards and Clinical Commissioning Groups being made accountable for Public Health commissioning in England from 2013, which informed an additional funding requirement for projects to address local needs and gain approval from Local Health and Well-being Boards in Round Two; a change which is reflected in the target populations and objectives of those projects.

### Objective Two: Influences on Evaluation Practices.

We identified five main themes describing factors that influenced evaluation practices: (1) programme and project design; (2) evaluation design; (3) partnerships; (4) resources; and (5) organisational structures and systems. Examples of how various factors within these themes can act as barriers or facilitators to evaluation are shown in Table 3, and explored further below. The data highlighted the complex inter-connections between influences, and how many influences can act as both facilitators and barriers depending on the project characteristics and context.

**Table 3:** Summary of influences on evaluation practice

Influence	Examples of how these can act as barriers or facilitators
**Programme and project design**	
Timescales	Lead in time, delivery and funding cycles influence opportunities for relationship building, recruitment, piloting methods and formative evaluation.
	Scheduling and duration of delivery sessions influence resource availability and capacity for data collection.
Participant demographics	Participant demographics influence recruitment and data collection, capacity for self reporting, response rates, outcomes of interest, requirements for different outcome measures and need for adaptations to data collection methods (impacts standardisation and generalisability).
Settings	Location, facilities and resource availability influence recruitment, response rates and data collection.
Implementation	Tailoring and adaptability in project and evaluation implementation can facilitate recruitment, participant engagement and response rates, but limit standardisation.
**Evaluation design**	
Standardised data collection	Facilitates consistency of reporting and comparability, however use in diverse project contexts and participant groups limits generalisability.
	Increases research-practice tensions, data collection burden and impacts response rates.
	Choice of tools, appropriateness to participants, and ease or difficulty of implementation influence data collection and outcomes.
Standard Evaluation Frameworks	Evaluation frameworks and guidance facilitate more consistent evaluation and reporting of required evaluation criteria and outcomes of interest.
	Variability in how criteria are applied and reported can act as a barrier to generalisability and quality of data.
	Limitations in guidance included in frameworks used can lead to variability in the quality of evaluation and reporting of specific evaluation components.
Use of non-required evaluation methods	Use of non-required evaluation components is dependent on knowledge, experience and priorities of project stakeholders, e.g. the value placed on qualitative methods influenced the inclusion of qualitative methods.
	Limitations in the specified requirements to address objectives drives inclusion of additional methods.
	Limitations in guidance, understanding of methods and capacity to conduct qualitative research influences the quality of analysis and reporting.
	Pilot and formative evaluation facilitates development, testing and embedding of evaluation approaches and data collection systems, intermediate evaluation facilitates learning, adaptation and improvement. These are dependent on timescales, regular
	reporting and feedback processes.
	Adaptability and flexibility facilitates ability to be responsive to needs, to improve participant and stakeholder engagement with evaluation processes, and to improve response rates and quality of data collection.
**Resources**	
Staffing	Staff expertise, experience, capacity, buy-in for evaluation, and how roles and responsibilities are defined influence evaluation processes, project sustainability, knowledge management and dissemination.
Funding level	Funding for evaluation, including staffing and partnership working, is a major influence on evaluation practice.
	Differing levels of funding and the proportion allocated to evaluation, position of decisions for this at local or national level, and timescales of funding cycles influence evaluation practices.
Time	Time impacts the choice of evaluation methods, and the capacity for data collection and evaluation processes.
Equipment/facilities	Influences project activities, recruitment, implementation, and data collection methods, including opportunities for use of innovative methods.
**Partnerships**	
Essential partners/roles and responsibilities	Definning roles and responsibilities of delivery, funding & evaluation partners for evaluation processes is a key factor.
	Capacity for evaluation and success of partnership working is dependent on costs, funding, resources, and the nature of the partnership.
Stakeholder priorities, objectives and expectations	Differing partner priorities and expectations can lead to research-practice tensions.
	Approaches to balance research objectives, policy priorities and practicalities of what will work in real-world & in budget are required.
	Strategies to manage expectations are needed.
Expertise, experience, capacity	Prior experience, knowledge and training of stakeholders influence evaluation design, choice of methods, innovation and implementation.
	Research-practice partnerships can improve evaluation through access to expertise, skills and experience, and access to additional resource for implementing evaluation and data collection.
Relationships and Communication	Close relationships between partners are key.
	Local partnerships increase opportunities to observe and understand local project needs and facilitate relationship building.
	Available, approachable and adaptable partners enable open and trusting relationships, regular comminication, opportunities for stakeholders to challenge, learn from each other, find solutions and make decisions collaboratively.
	Appropriate language facilitates relationship building (jargon busting).
History of partnership, embeddedness	Continuity of relationships facilitates understanding of local project evaluation priorities, helps to embed processes, which can help mitigate effects of limited lead-in times, piloting and insight phases.
	Arms-length or transactional relationships act as barriers.
**Organisational structures, systems and processes**	
Funding systems and requirements	Clearly defined, agreed and communicated funding requirements act as facilitators to evaluation and use of evidence.
	Funding cycles and time scales for reporting and review can limit learning from evaluation, dissemination and project sustainability.
	Understanding future commissioning needs facilitates evaluation planning and implementation to ensure practice-relevant evidence is collected.
Staffing structures	Clearly defining roles and responsibilities of staff, volunteers and partners is vital to successful partnership working, project implementation and evaluation processes.
	Key staff that have capacity &/or responsibility for co-ordinating processes, relationships and practices can be essential for the success of a project and its evaluation. These may be embedded in the staff structure as an evaluation officer, or an external partner that champions evaluation.
	Highly mobile workforce & employment contracts linked to short funding cycles act as a barrier to continuity of partnerships, relationships, and organisational learning, but as a facilitator to inter-organisational learning.
Systems for oversight, monitoring and communication	Information and support from funders, essential to guide project planning, but also to make use of feedback from intermediate monitoring and evaluation.
	Service level agreements help to define and agree roles, responsibilites, objectives and outputs, but can limit adaptability and flexibility.
	Steering groups (project boards or operational groups) enable sharing of good practice, open dialogue and support.
	Regular meetings that include evaluation feedback facilitates evaluation process. Challenges remain to ensure decisions are transferred between strategic and operational stakeholders, and that actions agreed are followed up.
Processes for capacity building and knowledge exchange	Training to build capacity, knowledge and gain buy-in is essential, especially where data collection is dependent on delivery staff.
	Workshops and networking opportunities facilitate knowledge exchange across projects, partners and wider audiences.
Data management systems	Effective data management systems facilitate data collection and management, participant engagement and project implementation.
	Developing, agreeing and embedding systems that meet the needs of practitioners and researchers is essential, but has implications for resources such as time, staffing and budgets.
	System development and use needs to consider implications for data security policies and practices, reliability, flexibility, integration with existing service delivery systems and needs, standardisation to allow reporting and comparison between partners, projects and programme.
Wider external influences	Embedding project and evaluation into existing service delivery offers opportunities for efficiencings, e.g. shared resources, staffing economies and use of existing infrastructure such as data management systems. Embedding in existing service delivery can also facilitate project sustainability.
	Evolving policies, strategies, commissioning priorities and knoweldge development interact to influence priorities for funding, project and evaluation objectives, reporting and desimmination, and use made of evidence.
	Multi-sectoral, multi-component projects or localised delivery and evaluation can lead to fragmentation of projects across organisations and locations, which can act as a barrier to standardised approaches to evaluaton, knowledge exchange and use of evidence.
Organisational culture and embeddedness of evaluation	Organisational culture and a history of evaluation and partnership working within organisations can increase opportunities for integrating evaluation and project design, improve the skills base, capacity and buy-in to evaluation process and practices and facilitate the embedding of evaluation.

#### Programme and project design

Evaluation was shaped by the programme and project design. The choice and use of evaluation and data collection methods within projects was determined by programme and project objectives and outcomes of interest. However, these also needed to be adapted to the contexts and characteristics of the projects. Within this theme we identified four sub-themes of important influences on evaluation: timescales, participant demographics, settings, and implementation.

Timescales were seen as a barrier to data collection and to formative work. For example, short lead-in times impacted participant recruitment, ability to pilot evaluation methods, and to develop and embed data collection systems. Stakeholders noted that it took time to build relationships with delivery partners and to recruit participants. Timescales related to funding, project conclusion and outcome review were also felt to be a barrier to project sustainability. For example, stakeholders commented:

“the main thing was that lead in time, and I think the second thing is that it takes time to set up the project especially in these hard to reach communities and I think you can't underestimate how much time it takes to build those relationships with the participants, community groups, with the referrers…so it is how we can move away from that two to three years funding cycle, with the reality that it probably takes a year to two years to build relationships in the community and then you are taking that intervention away.” (stakeholder 15)

“I think there was sometimes a lack of time to actually pilot test some of the data collection instruments and processes because the projects are under pressure to start delivering as quickly as possible. And if we had had that time we might have maybe done things differently or refined things before we actually started to ensure it all went smoothly.” (stakeholder 21)

Participant demographics also influenced the outcomes of interest and how data were collected. Stakeholders described the importance of adapting data collection methods, project design and activities, to facilitate recruitment and data collection with specific demographic groups.

Project locations, settings and contexts, including resource availability and accessibility for participants, further impacted recruitment, implementation and response rates. The need for flexibility and adaptability was a recurring theme. This was linked to changes to projects during implementation, such as: staffing and promotional material; adding or tailoring activities and engagement opportunities; and refining eligibility criteria or referal processes. Flexibility in both project and evaluation implementation were described as essential to facilitate data collection, whilst also being a potential barrier to the generalisability of outcomes.

#### Evaluation design

Evaluation design was shaped primarily by the requirements to use standardised data collection tools and a standard evaluation framework. In addition to these required elements, projects reported on a wide range of study designs, evaluation methods, and data collection tools, as shown in Table 4. As one stakeholder explained:

*“There was a big influence there in terms of consistency across the projects across the country … Sport England were a big influence in terms of the IPAQ and the things that they were asking for, but we also had the additional secondary questions that we added into the evaluation that were very much around what do we need locally to evidence that this works … I know that a lot of the academic studies included a process evaluation, but that wasn't a direct output that Sport England were expecting, or they didn't dictate that.”* (stakeholder 6)

To illustrate how the application and reporting of required and optional evaluation methods influenced the evaluation in practice these elements are discussed below.

**Table 4:** Study design and data collection methods included in project evaluation

Methods	Project Codes:	1-01	1-02	1-03	1-04	1-05	1-06	1-07	1-08	1-09	1-10	1-11	2-01	2-02	2-03	2-04	2-05	2-06	2-07	2-08	2-09	2-10	2-11	2-12	%
Physical Activity Measurement	International Physical Activity Questionnaire (IPAQ short)	x	x	x		x	x		x	x	x	x		x	x	x	x			x	x	x	x	x	78
	International Physical Activity Questionnaire (IPAQ-E)												x					x	x						13
	Scottish Physical Activity Questionnaire (SPAQ)							x																	4
	Stanford 7 day recall				x																				4
	Sport participation question (adapted from IPAQ)	x		x		x	x	x		x	x							x		x			x		43
	Objective measure using accelerometer in subsample				x						x		x									x			17
	Borg scale																							x	4
Screening	Single Item Measure	x		x	x	x	x		x		x		x	x	x	x		x		x		x	x	x	70
	Physical Activity Readiness Questionnaire (PARQ)							x				x					x				x				17
	General Practice Physical Activity Questionnaire (GPPAQ)		x																						4
Self-report Surveys	Cancer Physical Activity Standard Evaluation Framework (CaPASEF)							x																	4
	Health Related Quality of Life (EQ-5D-5L, EQ-5D-3L &/or VAS)	x		x				x	x				x	x				x							30
	Kemp Qulaity of Life Scale																		x						4
	Warwick Edinburgh Mental Wellbeing Scale (WEMWS)					x							x	x			x			x					22
	Functional Assessment of Chronic Illness Therapy (FACIT-Fatigue scale)							x																	4
	General Self-Efficacy (GSE) scale							x															x		9
	Wellbeing (e.g. Adolescent Wellbeing Scale, Well-Being Questionnaire)			x		x				x		x													17
	WHO-5 Well-being Index																						x		4
	RAND SF32		x									x													9
	Loneliness Questionnaire											x	x												9
	Motivation Questionnaire											x													4
	Fear of Falling Visual Analogue Scale												x												4
	Life satisfaction scale	x			x																				9
	Cantril Self-Anchoring Striving Scale															x									4
	Mediators of sport or physical activity (self report & other)		x			x												x			x	x			22
	Other self reporting (e.g. health status or behaviours)				x	x	x								x				x		x	x			30
	Feedback/satisfaction survey	x	x						x												x				17
Other	Attendance	x		x			x										x			x		x			26
	Costs, resource use, programme records	x		x				x	x				x	x				x		x			x		39
	Objective measures (e.g. anthropometric, health, functional fitness)		x										x				x			x	x				22
	Interviews, Focus groups	x	x	x		x	x	x		x	x		x	x	x	x	x	x	x	x		x	x	x	83
	Ethnographic/observation			x				x					x		x										17

##### Use of standardised tools

Sport England recommended using the Single Item Measure [35] to identify inactive participants for eligibility. Sixteen projects reported using this tool. Two projects did not refer to any screening tool, whilst four mentioned using alternative screening tools (Table 4). There was variability in how eligibility criteria were applied, and in the use made of the Single Item Measure; for example four projects used it to assess changes in physical activity over time. Stakeholders reflected on differences in how eligibity criteria and screening tools were applied as a challenge to recruitment and comparability across projects.

Projects were also required to use the IPAQ to collect baseline and follow-up measures. Twenty-two projects reported using IPAQ-short form or IPAQ-E (developed for older people), whilst one project had agreement to use an alternative tool, the Scottish Physical Activity Questionnaire (SPAQ). Sport England also recommended using a single question to assess sport participation; which ten projects referred to.

The use of standardised tools in real-world settings and with specific demographic groups was identified as a key challenge. In particular, stakeholders emphasised the negative effect of data collection burden on recruitment and response rates, and in turn on generalisability. For example, stakeholders described the following challenges in using the IPAQ:

“One of the biggest challenges is taking validated questions and looking at the practicality of implementing them in the community.” (stakeholder 15)

*“They were a fairly lengthy questionnaire for the type of people we were working with and it led to a real reduction in numbers. The evaluation led to the reduction in numbers. The reduction in numbers was because of the way the evaluation was working but to make the evaluation effective we needed more people. So it was a bit of a vicious circle.”* (stakeholder 19)

##### Use and reporting of the standard evaluation framework

The purpose of including the use of the essential SEF criteria as a funding requirement was to facilitate standardised evaluation and reporting. According to one programme-level stakeholder its strength was in the guidance on reporting contextual factors that would allow Sport England to *“understand what works, for who and how; or what doesn’t.” (stakeholder 1).*

Eleven (48%) of the evaluation reports, specifically stated that the evaluation was guided by the SEF. Eleven reports did not refer to any evaluation framework, and one referred to the RE-AIM framework [25] as guiding the evaluation.

Reporting of the SEF criteria was variable. Tables 5 and 6 summarise which projects reportedon the criteria related to programme details and participant demographics. All projects gave a detailed description of their aims and objectives, recruitment methods, location and setting, and reported on age and gender. Those that targeted specific population groups described these in detail. Quality assurance mechanisms, potential unintended consequences, and costs were reported on by fewer projects. The rationale for the intervention, relevant policy context and health needs assessment were not always differentiated. The SEF recommends the use of a logic model, yet just five reports (22%) provided this.

**Table 5:** Summary of project reporting on SEF criteria related to programme details

Project Codes:	1-01	1-02	1-03	1-04	1-05	1-06	1-07	1-08	1-09	1-10	1-11	2-01	2-02	2-03	2-04	2-05	2-06	2-07	2-08	2-09	2-10	2-11	2-12	%
SEF mentioned	X				X	X	X	X	X			X					X	X	X			X		48
1. Intervention title	X	X	X	X	X	X	X	X	X	X	X	X	X	X	X	X	X	X	X	X	X	X	X	100
2. Aims & objectives	X	X	X	X	X	X	X	X	X	X	X	X	X	X	X	X	X	X	X	X	X	X	X	100
3. Rationale for the intervention	X	X	X			X	X	X	X	X	X	X			X	X	X	X	X	X	X	X	X	83
4. Contact details	X	X	X		X	X	X		X		X	X	X						X		X	X		57
5. Commissioners & sources of funding	X	X	X		X	X	X	X	X	X	X	X	X	X	X	X	X	X	X	X	X	X	X	96
6. Intervention timescale	X	X	X		X	X	X	X	X	X	X	X	X	X			X	X	X	X	X	X	X	87
7. &/or 8. Delivery or funding dates	X		X		X	X	X	X	X	X		X	X	X	X	X	X	X	X		X	X	X	83
9. Location & setting	X	X	X	X	X	X	X	X	X	X	X	X	X	X	X	X	X	X	X	X	X	X	X	100
10a. Target population	X	X	X	X	X	X	X	X	X	X	X	X	X	X	X	X	X	X	X		X		X	91
10b. Content	X	X	X		X	X	X	X	X	X	X	X	X	X	X	X		X		X	X	X	X	87
10c. Delivery method	X	X	X		X	X	X	X	X	X	X	X	X	X	X	X	X		X	X	X	X	X	91
10d. Deliverer	X	X	X	X	X	X	X	X	X	X	X	X	X	X	X		X	X		X	X	X	X	91
10e. Quality assurance mechanisms							X	X	X	X			X			X					X			30
10f. Potential unintended consequences			X						X															9
11. Method of recruitment & referral	X	X	X	X	X	X	X	X	X	X	X	X	X	X	X	X	X	X	X	X	X	X	X	100
12. Admission/inclusion criteria	X	X	X		X	X		X	X	X			X	X		X			X			X	X	61
13. Consent mechanism/ethical approval	X	X	X		X	X	X		X	X		X			X	X	X	X	X		X	X	X	74
14. Equipment & resources	X		X		X	X	X		X	X		X	X				X		X					48
15. Core staff competencies/training	X		X		X	X	X	X	X	X		X	X	X	X	X	X		X		X		X	74
16. Incentives for attendance	X		X	X	X	X	X	X	X	X	X		X					X			X			57
17. Detailed breakdown of costs	X		X				X	X				X	X				X		X			X		39
18. Costs per participant	X						X	X		X		X	X						X		X	X		39
19. Cost to the participant	X	X			X	X	X		X	X	X		X		X		X	X						52
20. Relevant policy context	X		X				X	X	X	X	X	X	X		X	X	X	X	X	X	X		X	74
21. Health needs assessment			X	X		X		X					X			X	X		X	X		X	X	48
22. Equality impact assessments																								0
23. Declaration of interest																								0

**Table 6:** Summary of project reporting on SEF criteria related to participant demographics

Participant Demographics	1-01	1-02	1-03	1-04	1-05	1-06	1-07	1-08	1-09	1-10	1-11	2-01	2-02	2-03	2-04	2-05	2-06	2-07	2-08	2-09	2-10	2-11	2-12	%
Age	x	x	x	x	x	x	x	x	x	x	x	x	x	x	x	x	x	x	x	x	x	x	x	100
Sex	x	x	x	x	x	x	x	x	x	x	x	x	x	x	x	x	x	x	x	x	x	x	x	100
Ethnicity	x	x	x	x	x	x	x	x	x	x	x	x	x	x	x	x	x		x		x	x	x	91
Disability	x	x	x	x	x	x	x	x	x	x	x	x		x	x				x		x	x	x	78
Socio-economic status	x	x	x	x	x	x	x	x	x		x		x	x	x		x	x	x		x	x	x	83
Additional information e.g. health status	x	x	x		x	x	x				x								x			x		39

All projects reported on the timing of data collection at baseline and follow-up. Whilst there was some variation in how impact data were reported, all projects reported on change in self-reported physical activity across time points. Seven (30%) projects reported a comparison of outcomes between intervention and control groups or across demographic, disease-risk, referral or service pathway sub-samples. Details of statistical tests used to analyse physical activity measures and the rationale for their use were reported fully, whilstsixteen (70%) projects reported on limitations and generalisability and ten (44%) reported on how findings were disseminated.

The SEF provides more limited guidance on process evaluation (Table 1). Participant numbers were reported variably based on attendance at at least one session, completion of a 10 or 12 week course, or registration at one-off events or online. One project provided a flow diagram of participant numbers with reasons for drop out. Fourteen (61%) projects combined exit survey and interview data to report on participant satisfaction. Nineteen (83%) projects reported on plans for sustainability. One project included this as a research objective to explore features that may lead to sustainable delivery models. Five (22%) projects described how the delivery model had been developed with sustainability in mind.

##### Use and reporting of optional evaluation components

Table 4 shows that projects included a range of additional self-report surveys. Nineteen (83%) of the projects conducted interviews and/or focus groups to provide additional understanding and insights about how the projects worked and were received. The choice and use of these methods was influenced by project level stakeholders’ priorities and expertise, but also limitations in the required tools to generate evidence in relation to evaluation objectives.

Several stakeholders reflected on the value of qualitative methods to answer questions about the project, for example:

*“there's certain cohorts of people we work with where it’s really hard to collect robust evaluation and actually it's the qualitative that matters and the process. I'd like to see a lot more investment in process evaluation because I think at the moment at this time of system changes, so much transformation going on in the health system, and it’s the processes that are important.”* (stakeholder 6)

“*I think for us some of the most important information came from the qualitative side.*” (stakeholder 15)

Twelve projects provided a separate section or report described as either a process or qualitative evaluation. There was variability in how qualitative methods were applied, analysed and reported. For example, some simply mentioned thematic analysis, whilst others provided details of the coding and method of reporting. Four projects combined different data sources to explore project impementation and contextual factors, whilst eight reported on data as case studies of individual participants, organisations or delivery pathways.

#### Resources

Resources, including staff, time, funding, equipment and facilities, were a major influence on evaluation as shown in Table 3. In particular, the availability and use of resources illustrates how the context and characteristics of each project can affect how factors interact and can act as both facilitators and barriers. For example staffing was essential for data collection and evaluation, and depended on the roles, responsibilities and capacity of partners, which in turn were dependent on organisational staffing structures, funding levels and time-scales. Stakeholders from some projects regarded the level of funding as enabling a more rigorous evaluation process than is often possible within real-world interventions, whilst stakeholders from other projects highlighted limited funding as a barrier to their ability to resource the evaluation.

#### Partnerships

Partnerships shaped the nature of project evaluations. All projects were required to have an independent evaluation partner, and were developed and implemented through working with a range of delivery and funding partners. Evaluation partners were central to the evaluation design. Whilst some stakeholders reflected on differing objectives, priorities and understanding between research and practice as potential sources of tension, most highlighted access to expertise, and in some cases access to additional resources for evaluation as a benefit.

Variation in the responsibilities, priorities and capacities of staff employed by delivery organisations and evaluation partners was thought to have impacted the evaluation design and process. Delivery staff were seen as essential to recruitment and managing data collection. Defining responsibilities, communication, and training were seen as vital to build capacity,and to get buy-in to the evaluation process. As shown in Table 3, the nature of the relationships and history of the partnerships were key influences. For example, close relationships and local partnerships enabled regular communication, and facilitated relationship building and sustainable partnerships, whereas arms-length relationships were described as barriers to successful partnerships and evaluation.

#### Organisational structures, systems and processes

We identified seven sub-themes of influences related to organisational structures, systems and processes: funding systems; staffing structures; systems for communication, monitoring and oversight; processes for capacity building and knowledge exchange; data management systems; wider external influences; and organisational culture and embeddedness of evaluation (Table 3).

Several of these factors are inter-connected, and also underpin factors identifed within the other main themes. For example, whilst defining roles and responsibilities early in the project was essential to successful partnership working and evaluation, this was dependent on appropriate funding and staffing structures. High staff turnover was mentioned as a challenge to evaluation in nine of the reports, and by eighteen of the stakeholders interviewed. Stakeholders felt this was linked to short funding cycles and contracts, and to have negatively influenced continuity, the capacity for evaluation and dissemination. In particular, stakeholders felt that delays in staff recruitment added to the challenges associated with short lead in times; and early departure of staff influenced dissemination and use of evidence. Having a central co-ordinator who could act as a conduit between partner organisations was seen as critical to successful project evaluation in several cases.

As shown in Table 3, various structures and systems that can act as facilitators to evaluation were identified. Examples include: steering groups and service level agreements to enable regular and formal communication and oversight; training and knowledge exchange to build capacity; and data management systems and processes to integrate evaluation within normal service delivery. Stakeholders reflected on the potential for efficiencies from integrated systems and processes, but also on the considerable time and resource implications of developing these and the difficulties in implementing them across multiple project partners and/or components.

A key underpinning theme was the importance of systems to facilitate monitoring, oversight and communication throughout the project planning, implementation and evaluation cycle. However stakeholders reflections on their experiences of these were variable. For example, service level agreements were seen as critical to agreeing and defining responsibilities in some projects, and as limiting flexibility in others. Many stakeholders reflected on the value of networking and knowledge exchange events facilitated by the funding agency, whilst others commented on a lack of such oportunities as a limitation:

*“We found the workshops that they held, … actually to get the GHGA projects in a room together was really useful and because you could share the issues that you were having and people understood and you could share ideas and realize how people have overcome them.”* (stakeholder 24)

*“They were really good at that side of things, they would bring us in and then different projects would speak each time on different topic areas that we would cover in workshop scenarios, that was really good. They did that really well … I think Sport England could make a lot more of the network than they do in terms of avoiding that duplication of effort and resources*.” (stakeholder 6)

*“I never had a chance to talk to anyone else who was doing any of the other evaluations so there was never that kind of network and support which I think it might have been quite useful to have had*.” (stakeholder 28)

Variability in communication and involvement of stakeholders in networking across different projects appears to have limited the opportunity for a more consistent approach to wider scale knowledge exchange and use of evidence. Some stakeholders also identified a need for organisational structures that enabled forward planning and closer working with local services to ensure that evaluation and evidence generation met future commissioning requirements.

### Objective 3: appraisal of whether the programme was effective in generating high quality generalisable evidence that enabled it to meet its aims

**Fig. 3:**
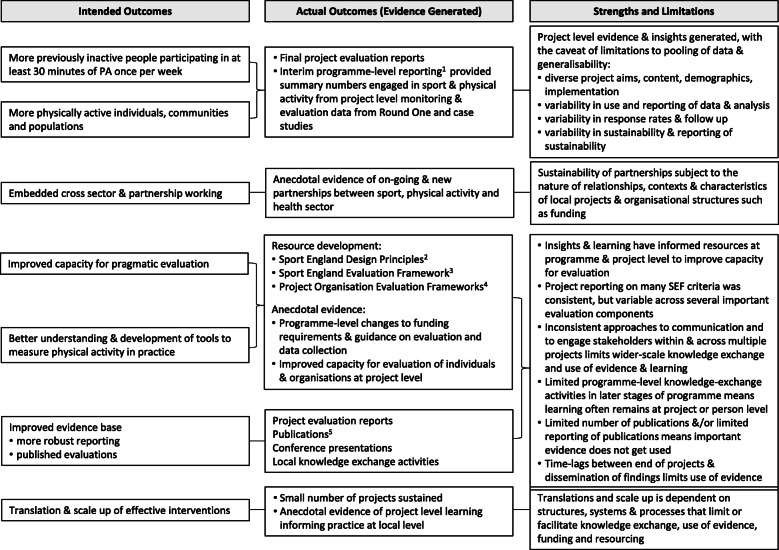
Evidence generated from the Get Healthy Get Active programme mapped against the intended outcomes. Notes:1*Get Active Get Healthy, what we have learned so far* [34], *Tackling Inactivity* [43]*, 2Design Principles* [44]*, 3Sport England Evaluation Framework* [45]*, 4Hertfordshire Evaluation Framework* [49], 5Examples of publications include [50–60]

Figure 3 provides a summary of project and programme outputs mapped against the intended outcomes included in the logic model (Fig. 1). Two separate evaluation consultancies were commissioned to produce summary reports from Round One and Round Two respectively. At the time of writing, only the reports following Round One were available [34, 43]; these reported numbers of participants engaged in the programme, changes in numbers of participants identified as active or inactive, and case studies of individual projects. Stakeholders at programme and project levels acknowledged the challenges of pooling large data sets from multi-component, multi-sectoral projects due to diverse project designs, settings and participant demographics, and variability in response rates, secondary outcomes, and in how outcome measures were analysed and reported:

*“It was good to specify a measure to get the consistency across all the programmes, I guess the quality of that data collection probably varied quite a lot across different projects, depending on who did the data collection and how it was done.”* (stakeholder 21)

One programme level stakeholder commented on the need to accept flexibility in how projects applied the specified requirements but that this:

“*created a number of challenges at programme level, when you try to pull it all together.”* (stakeholder 1)

Programme level stakeholders reported that findings had informed the development of resources to support project and service design and evaluation [44–46], and that several project reports had been included in subsequent reviews of practice [47, 48]. In total nine projects disseminated findings through published articles in academic journals, eleven through publicly available reports, and nine through conference presentations. Five stakeholders mentioned plans for publishing articles, but identified a lack of time or time lag between end of project and publication as a challenge.

Project level stakeholders felt the need for knowledge exchange activities and reporting methods that were more appropriate to a wider audience, including local stakeholders and commissioners. Stakeholders involved in projects that had been showcased through best practice projects and conferences saw it as an important way of valueing the project and disseminating findings. Other stakeholders, who had not been involved seemed less aware of dissemination activities beyond what they were doing locally, and were keen to know more about how findings from across the programme were being shared. For example, stakeholders commented:

*“I think it is a constant frustration that I have, that there is a huge amount of knowledge that gets built up and then never gets shared.” (*stakeholder 31)

*“I don't think out of all those projects across the whole network, that was really shared with people. So I think we got to hear more about it because we were part of it. I think where they have done one or two things more recently where they do try and bring people back together where they are all working on similar types of project and I think that's really valuable but I still think they can do a lot more to then share that with the wider network.” (*stakeholder 30)

Whilst there was limited understanding amongst some project level stakeholders of how the reports were received, used or shared at the programme level, many described project evaluation as influencing practices, project sustainability or partnerships locally. One programme-level stakeholder commented on learning and capacity building remaining at a project or person level, and fragmentation of projects across multiple organisations, limiting the ability to influence at scale.

## Discussion

The GHGA programme included physical activity projects with a wide range of secondary aims, partnerships, participant groups, settings, and project and evaluation designs. Despite the variability in projects, we identified common influences on evaluation practices that act as facilitators or barriers depending on the context and how they interact within a project. Multiple factors influence programme implementation and evaluation in real-world interventions [16, 19]. This is especially true in multi-sectoral and multi-component programmes such as GHGA. This makes gauging the role of any one factor difficult. Accordingly, our findings highlight the importance of understanding the interactions between influences on evaluation practices and, in particular, the implications for commissioning and evaluation of interventions. Whilst our focus is on physical activity interventions, the findings are applicable to other interventions, particularly those operating in multi-agency public health contexts.

A frequent criticism of real world evaluation has been that evaluation is approached as an “add on” to intervention design and implementation, and that insufficient attention is given to evaluation during intervention planning [7, 16]. Previous studies of health promotion programmes have also identified barriers such as limited investment for evaluation, and differing value placed on evaluation by stakeholders [7, 8, 61, 62]. Within the GHGA programme these barriers were largely overcome by the specification of evaluation as a funding requirement at the outset of the programme. Our study showed the vital role that commissioners play in influencing evaluation practice through resourcing and demands for evaluation, and more critically, in providing appropriate guidance and support, and how they value different forms of evidence.

Stakeholders’ understanding of what counts as evidence, and their use of appropriate evaluation methods, are recognised challenges of conducting real-world evaluation [8, 63–66]. Evaluation in an applied context often requires a balance to be found between scientific rigour and pragmatism, internal and external validity, and standardisation and adaptability [8, 22]. It can be a challenge to balance differing stakeholder priorities for evidence. The value of combining systematic and flexible approaches [67–69], and applying theory based approaches [20, 21, 70] to evaluate the variability within complex interventions is well recognised. Standardised requirements for evaluation of funded projects can facilitate a systematic approach to evaluation and improve the consistency of reporting. This may be particularly important within multi-project programmes like GHGA, which are designed and funded nationally but delivered and evaluated through local projects. We have previously argued that appropriate use of an evaluation framework to guide evaluation and reporting can improve the quality of an evaluation study [28]. Use of a framework can also facilitate identification and agreement of evaluation objectives and methods between stakeholders [71]. Logic models are commonly recommended to identify objectives, inputs, contextual factors and outcomes to help explain an intervention’s theory or rationale [22, 24, 72, 73]; their use is also recommended in the SEF [26]. Qualitative or mixed methods are also advocated to help explain quantitative findings, and generate evidence about project implementation, programme theory or causal mechanisms [14, 24, 29, 69]. Despite putting in place specific evaluation requirements, there was considerable variation in how important evaluation components were applied and reported. Components that were reported in detail, such as project descriptions and participant demographics, reflected the more detailed guidance of these components in the evaluation framework applied. Gaps in the evaluation reports highlighted limitations in the guidance provided in the SEF and the field generally on important evaluation components, and limited the ability to compare or generalise findings across projects. Further guidance or training is needed to improve the evaluation and reporting of specific components, in particular qualitative methods, process evaluation, economic evaluation, logic models, and data analysis. We argue that specifying evaluation requirements alone is insufficient. The context-specific nature of influences within diverse projects makes it more critical to implement processes that facilitate collaborative decision making to select, agree and apply the most appropriate methods to generate the evidence required and valued, rather than specifying standardised data collection across heterogenous projects.

Evaluation partnerships were a strong influence on evaluation. Many of the benefits of partnership working that we identified in this study, such as access to expertise, capacity building, and efficiencies from shared resources or integrated systems were also found in other studies [7, 12, 16, 19]. We also suggest that partnerships can bring greater opportunities for evaluation to be tailored to the needs of individual projects and stakeholders, and to enable a more flexible and innovative evaluation approach. However, the effectiveness of partnerships were dependent on the nature of the relationships, the embeddedness and continuity of partnerships, and on organisational structures and systems. In line with other studies, we also found partnerships to be context specific, and changeable [50]. For funders and partners to initiate and embed processes and systems that facilitate partnerships and that retain benefits of partnership working beyond a projects lifetime, it is essential that we develop a better understanding of the influences of, and on, partnership working.

Our appraisal of the extent to which the programme had generated evidence to achieve its aims (Fig. 3) identified several resources and publications resulting from the programme, but showed that dissemination and use of evidence remains a challenge. At this stage, questions remain as to how useful local project evaluation has been in addressing the programme aim to build an evidence-base that would inform scale up of effective interventions or translation to other settings. The programme sits within a system of evolving national and local policies, strategies and priorities, and knowledge base (Fig. 2). Our findings highlight the importance of rapid feedback to ensure that evidence and insights are disseminated and used to inform policy and practice. Further, we show the importance of thinking forward to the next cycle of project planning and funding to ensure that relevant evidence is generated and used beyond the project. Systems that enable collaboration in the early stages of evaluation planning to identify and agree types of evidence needed and stakeholder engagement throughout the project lifespan are essential. In additition, systems are needed that minimise time lags between project end and dissemination and facilitate knowledge transfer between and beyond projects and partners. The role of research partners is critical in bringing practice-relevant studies to publication [12], and reviewers and editors also have a role in this. Our study showed that funders and practitioners have a vital role in facilitating and contributing to knowledge-exchange activities. Multi-sectoral and multi-component projects, particularly where projects and evaluation are locally designed and implemented, need appropriate processes and systems to facilitate flows of information between all stakeholders. Without this, fragmentation of projects can lead to fragmentation of learning across organisations and individual stakeholders. In line with other studies [16, 18, 19], we show that cross-sector partnerships and networks appear to offer opportunites to improve knowledge-management and dissemination. Further research is needed to understand their value and how these can be implemented and embeded to help close current gaps in the evidence-based practice cycle.

Our findings have highlighted the important influences of differing stakeholder demands for evaluation, and resources for evaluation, in shaping the design and implementation of intervention evaluation. More critically, it showed the important influence of the underpinning organisational structures and systems, and the complex interactions between influences that act as facilitators or barriers to good practice, even when measures to address known challenges are put in place. Previous studies have identified a need for multi-level strategies to improve evaluation and for more research to understand these [16, 19]; this study supports this view. We argue that stakeholders need to work together to understand, develop and implement systems to enable: (i) collaborative decision making; (ii) synergies between data needed for project delivery, participant engagement, accountability, research and evaluation; and (iii) timely knowledge transfer and dissemination. It is vital to improve our understanding of how influences interact to facilitate or limit good practice within evaluation. This will enable structures and systems to be developed and implemented that capitalise on factors acting as facilitators and that address barriers, and help to ensure that effective interventions are adopted, and that ineffective interventions or unnecessary research are avoided.

### Strengths and limitations

A key strength of this study is that we combined data from multiple sources, including evaluation reports and documents from 23 physical activity projects and from the programme as a whole, and data from 35 stakeholder interviews. A further strength is our use of a rigorous and transparent methodology to extract and analyse the data. The logic model that we imputed from the documents was based on the programme aims, objectives and intended outputs reported, and implied outcomes, and was further refined through consultation and interviews with key stakeholders.

There are several limitations of the study. Time lags between end of project delivery and publication mean that our appraisal of the evidence generated could not include the final programme summary evaluation that has been commissioned, and we may have missed additional publications from individual projects. The retrospective nature of the study limited the use of a more ethnographic approach. This may also have contributed to a lower response rate from project organisations and our ability to obtain documents related to project planning and the funding application. This time line also limited our ability to adopt a more collaborative approach to agree the theory of the programme as represented on the logic model.

## Conclusion

We identified multiple influences on evaluation practice that can act as barriers and facilitators to good practice. These influences are context-specific and operate through a complex set of interactions. It is vital that commissioners, researchers and practitioners engaged in intervention evaluation or with an interest in improving evaluation and the generation of high-quality evidence, develop a better understanding of these influences and implement appropriate systems and processes to support good practice. Critically, organisational structures, systems and processes are needed to: (i) build and retain individual and organisational capacity for evaluation; (ii) enable collaborative and flexible decision making to identify and agree the most appropriate evaluation objectives, methods and types of evidence; and (iii) improve the transfer of knowledge and insights between stakeholders. This is critical to close current gaps in the evidence-based practice cycle, and ensure that relevant evidence is generated and used in a timely manner.

## Supplementary Information

**Additional file 1.** Interview Guide

## Data Availability

Documents used to support the findings of this study are publicly available. Other dataset(s) used and analysed during the current study are not publicly available due to them containing information that could compromise research participant consent and anonymity. Data sets are available from the corresponding author on reasonable request, and subject to permission from Sport England.

## Abbreviations

BMI: Body mass index
CCG: Clinical commissioning group
GP: General practitioner
GHGA: Get healthy get active
IPAQ: International physical activity questionnaire
PA: Physical activity
RCT: Randomised controlled trial
RE-AIM: Reach, effectiveness, adoption, implementation, maintenance framework
SEF: Standard evaluation framework for physical activity interventions
SPAQ: Scottish physical activity questionnaire
WHO: World health Organization
