# Supplementary material for: Exploring influences on evaluation practice: a case study of a national physical activity programme
Source: Int J Behav Nutr Phys Act. 2021 Feb 16;18:31. doi: 10.1186/s12966-021-01098-8 (PMC7885395; doi:10.1186/s12966-021-01098-8)
Supplement: Supplementary file 1 — Additional file 1. Interview Guide [file 12966_2021_1098_MOESM1_ESM.docx]

| **General Contextual**  1. Please can you tell me about your role in the project ? |
| --- |
| **Project Evaluation**  2. Can you tell me about your experiences of being involved in the project evaluation, please?  3. What do you feel were the main factors that influenced how the evaluation was designed and implemented?  Prompts: Any specific requirements, evidence, tools, or frameworks?  4. What do you feel were the main strengths and weaknesses of the methods or approaches used in the project evaluation?  Prompts: What worked well?/ What worked less well, any challenges?/ Any examples?  5. How useful do you feel the evaluation was?  Prompts: Any examples of how it was used?/ Any examples of challenges to it being useful?  6. Please could you tell me more about any systems or organisational structures that were put in place to support project evaluation? How effective were these?  7. Reflecting back, is there anything you feel you would have been done differently in evaluating the project?  8. Do you have any thoughts or suggestions for what is needed to support project evaluation? |
| **Partnership working:**  9. Please can you tell me more about any partners involved in the evaluation, and the roles they played?  Prompts: Who do you see as essential partners? / Had you worked together before? Who did what? How was this decided?  10. Can you tell me more about your experiences of working with partners as part of the evaluation?  Prompts: What works well? (facilitators) / What works less well? (any barriers or challenges?) / Anything you would do differently regarding partnership working?  11. How would you describe the processes, information or support for evaluation from partners?  Prompts: From the funder, within your organisation, other partners? |
| **Evaluation reporting/knowledge sharing:**  12. Please can you tell me more about your experiences of how the evaluations were reported or shared?  13. Do you have any thoughts on how projects could be better supported to share knowledge gained from evaluation? |
| **Other questions** e.g. Specific follow up on observations from the evaluation report. Is there anything else you would like to tell me, that you feel you have not yet had the opportunity to discuss? Is there anyone else involved in the project and/or it’s evaluation that you feel it may be useful for me to speak to? |
